# Cytotoxicity Potential of Endophytic Fungi Extracts from *Terminalia catappa* against Human Cervical Cancer Cells

**DOI:** 10.1155/2020/8871152

**Published:** 2020-09-22

**Authors:** Michèle Stella Majoumouo, Marius Belmondo Tincho, Rufin Marie Kouipou Toghueo, Thureyah Morris, Donavon Charles Hiss, Fabrice Fekam Boyom, Chitra Mandal

**Affiliations:** ^1^Antimicrobial Agents Unit, Laboratory for Phytobiochemistry and Medicinal Plants Studies, Department of Biochemistry, P.O. Box 812, University of Yaoundé 1, Yaounde, Cameroon; ^2^Cancer Biology and Inflammatory Disorder Division, Council of Scientific and Industrial Research-Indian Institute of Chemical Biology, 4, Raja S.C. Mallick Road, Kolkata 700032, West Bengal, India; ^3^Department of Biotechnology, Faculty of Natural Sciences, University of the Western Cape, Private Bag X17, Bellville 7535, South Africa; ^4^Division of Medical Virology, Department of Pathology, Faculty of Health Sciences, University of Cape Town, Cape Town 7925, South Africa; ^5^Food Toxicology Laboratory, Department of Medical Bioscience, Faculty of Natural Sciences, University of the Western Cape, Private Bag X17, Bellville 7535, South Africa; ^6^Molecular Oncology Laboratory, Department of Medical Bioscience, Faculty of Natural Sciences, University of the Western Cape, Private Bag X17, Bellville 7535, South Africa

## Abstract

Endophytic fungi are potential sources of novel bioactive metabolites from a natural product drug discovery perspective. This study reports the bioactivity-directed fractionation of the secondary metabolites of the ethyl acetate extract of a fermentation culture of endophytic fungi from *Terminalia catappa* which were then evaluated for their cytotoxicity against human cervical cancer (HeLa) cells and human foreskin fibroblast (HFF) cells. Furthermore, apoptosis was determined using the Annexin V/propidium iodide (PI) flow cytometry assay. Endophyte extracts N2, N7, N8, N97, N169, and N233 were obtained from *Trichoderma sp*, *Phoma sp*, *Phomopsis phyllanticola*, *Fusarium oxyporum*, *Collectotrichum sp*, and *Cryptococcus flavescens*, respectively. The N97 extract was most active with a 50% inhibitory concentration (IC_50_) of 33.35 *µ*g/ml. A 50% cytotoxic concentration (CC_50_) of 268.4 *µ*g/ml was obtained with HFF cells and the selectivity index (SI) was 8.01. The percentages of cell populations were increased at late apoptosis (Annexin+/PI+), with the percentages of 27.4 ± 0.3 and 19.2 ± 0.01 obtained, respectively, for 50 *µ*g/ml and 80 *µ*g/ml of the N97 extract and 2.1 ± 0.1 obtained for the control in late apoptosis (Annexin V+/PI+) . Moreover, a higher reduction in the percentage of viable cells was observed in the HeLa control cells (93.6 ± 0.3), but the percentages of viable HeLa cells were 37 ± 0.05 and 45 ± 0.1, respectively, for the 50 *µ*g/ml and 80 *µ*g/ml treatments with the N97 extract. Also, the percentages of 34.7 ± 0.1 and 33.9 ± 0.4 were, respectively, obtained for 50 *µ*g/ml and 80 *µ*g/ml compared to the control with 4.6 ± 0.2, in early apoptosis (Annexin V+/PI-). These findings highlight the anticancer potential of the N97 extract of endophytic fungi from *Terminalia catappa*, which is mediated through apoptosis and presumably also attenuation of chemoresistance.

## 1. Introduction

Despite the immense advances in medical sciences in recent years, over 32.6 million people are living with cancer worldwide. Cancer was once thought to be a disease prevalent in developed nations, but the disease is a global health problem. Recent statistics show that 65% (5.3 million) of all cancer-related deaths were reported from less developed countries [1]. Among these cancers, uterine cervical cancer is the fourth most common cancer diagnosed in women and the second leading cause of cancer-related morbidity in women worldwide [2]. In 2012, an estimated 528,000 new cases were reported resulting in 266,000 deaths [3]. According to recent reports [2], human papillomavirus (HPV) infections contribute to the high incidence of cervical cancer cases in low- to middle-income countries [2]. Anticancer drug toxicity and side effects such as nephrotoxicity, nausea, hair loss, skin irritation, anaemia, infertility, and resistance pose major challenges for successful cancer clinical outcomes [4, 5]. One way of overcoming such challenges is the identification of underexplored and/or unexplored biological sources to produce novel, efficacious anticancer compounds.

Endophytes are symbiotic fungi that receive shelter and nutrients from their host, while the host plant benefits from an array of attributes, including defense against natural enemies such as pathogens and herbivores [6]. The fungal endophytes inhabit host tissues in different organs, including leaves, stems, bark, roots, fruits, flowers, and seeds [7]. Traditionally, endophytic fungi have been considered as plant mutualists, benign commensals, or latent pathogens and are currently viewed as prime sources of bioactive natural products [8]. The intrinsic nature of the interactions among endophytes, host plants, and pests, which are mediated by bioactive compounds, is a paradigm for future discovery of sources of anticancer drugs [9].

Numerous studies have reported that medicinal plants from Cameroon with an ethnobotanical history are known to harbour endophytic fungi, which are considered as rich sources of novel bioactive products with antimicrobial, insecticidal, and anticancer activities [9]. Historically, these plants have served as important sources of medicinal products for the treatment of human malignancies [10–12]. However, only a limited number of plant species have been studied for the potential of their endophytes to produce bioactive compounds and very few surveys of endophytes from Cameroonian medicinal plants have been done [13].

In Cameroon, decoctions and infusions of the leaves, bark, and wood of the genus *Terminalia catappa* and *Terminalia mantaly* (*Combretaceae*) are used routinely for the treatment of several diseases such as gastroenteritis, dysentery, hypertension, diarrhoea, diabetes, headache, colic, intestinal parasites, oral, and skin conditions [14]. Another commonly used source of endophytic fungi is the plant *Cananga odorata* (*Annonaceae*), which is used to treat hepatitis and has a wide range of other medicinal applications [15]. The endophytic fungi from these plants constitute a credible source for the discovery of novel anticancer drugs. Therefore, this study aimed to narrow this gap in knowledge by screening the biological activities of a medicinal plant indigenous to Cameroon. Here we report the inhibition of HeLa cell proliferation, a hallmark of the cancer phenotype, by inducing apoptosis mediated by endophytic fungal extracts of *Terminalia catappa*. The cytotoxic effects of the N97 extract were evaluated on nontumour HFF cells to determine the possible differences in response to the extracts between cancer and normal cells.

## 2. Materials and Methods

### 2.1. Plant Material

The bark, flowers, leaves, and stem bark of healthy and mature plant organs of *T. catappa* (51244/HNC), *T. mantaly* (64212/HNC), and *C. odorata* (42250/HNC) were collected in Yaoundé, Cameroon (latitude 3°42′ N, longitude 11°20′ E), on September 2, 2014. All plant materials were directly brought to the laboratory in sterile bags and processed within a few hours after sampling.

### 2.2. Fungi Isolation

The endophytes (*Trichoderma* and *Fusarium* species) were isolated and the morphology was identified as previously described [16]. In brief, the plant material was rinsed gently in running water to remove dust and debris. The bark, flowers, twigs, leaves, stem bark, root, and root bark samples were cut into small pieces of ∼5 mm length after proper washing. The leaves were divided into leaf blade and leaf vein. The leaf blade, leaf vein, twigs, and stem pieces were washed in tubes containing a solution of 20% commercial bleach (1% active chloride) for 10 min. Bark, root, and root bark fragments were surface-disinfected by rinsing with 70% ethanol for 5 min, followed by treatment with a 1% active chloride solution for 15 min, 2 min in 70% ethanol, and a final rinse in sterile distilled water. The different plant parts were plated on Petri dishes coated with potato dextrose agar (PDA) medium (Sigma-Aldrich, St. Louis, MO) supplemented with chloramphenicol (200 mg/ml) and kept in the dark, at room temperature (22°C–26°C). Isolated mycelial fragments from the cultures were subcultured on new PDA-coated Petri dishes until pure cultures were visible. Pure cultures were maintained under natural light at room temperature. Sterility controls consisting of imprints of plant fragments cultured on PDA were periodically observed to determine if fungi emerged from the imprints [17].

The infection frequency of each plant part was calculated using the formula [18]

(1) $$\begin{array}{c} \text{infection frequency}\left(\%\right)=\frac{\text{number of fragments colonized by endophytic fungi}}{\text{number of fragments transferred by the culture medium}}\times 100\%. \end{array}$$

### 2.3. Fermentation of Isolated Pure Strains

Fermentation of isolated pure fungal strains was carried out in 500 ml flasks containing sterilized rice medium as previously described [19]. In brief, pure fungal strains (1-2 weeks growth on PDA) were cut into pieces and inoculated into a flask containing 100 g sterilized solid rice medium. A flask of rice medium without any inoculum served as a control or some of them were cultured in PDA broth. Cultivation was performed at room temperature under static conditions for 20–30 days (depending on fungal growth) and examined periodically for possible contamination.

### 2.4. Extraction of Metabolites from Endophytic Fungi

After the incubation period, the fermentation process was terminated with the addition of 300 ml ethyl acetate (EtOAc) to each culture flask. The cultures were then cut into pieces with the aid of a glass rod. The culture flask was then placed on a shaker for 48 h to allow complete extraction. The mixture was vacuum-filtered using a Büchner funnel followed by exhaustive extraction with EtOAc. The resulting extract was separated using liquid partitioning. The EtOAc extract was concentrated using a rotary evaporator, and the extracts were dried and kept at 4°C for further analysis.

### 2.5. Cell Lines and Culture Media

The human HeLa cervical cancer cell line and mammalian cell HFF (human foreskin fibroblasts) were purchased from the American Type Culture Collection (ATCC) (Manassas, Virginia, USA). The cells were grown in the DMEM medium supplemented with 10% heat-inactivated foetal bovine serum (HIFBS) (Sigma-Aldrich, St. Louis, MO), phenol red (Thermo Scientific, New Delhi, India), and 1% penicillin-streptomycin (Sigma, New Delhi, India) and were maintained at 37°C in an atmosphere of 5% CO_2_ and 90% relative air.

### 2.6. Cytotoxicity Effect of the Extracts against HeLa Cell Line

The cytotoxicity of the extracts on HeLa cells was performed using the 3-(4, 5-dimethylthiazol-2-yl)-2, 5-diphenyl tetrazolium (MTT) cell proliferation assay as previously described [20]. Cells at 80% confluence were detached and harvested by trypsinization and then seeded at a density of 7 × 10^3^ cells/well and 100 *μ*l of cell suspension was added to each 96-well plate and grown to confluence at 37°C and 5% CO_2_. Stock solutions of the extracts (10 mg/ml) were prepared in 10% DMSO and serially diluted (0–500 *μ*g/ml) and added to the 96-well plate containing the cell line. The plates were incubated for 48 h at 37°C in an atmosphere of 5% CO_2_ incubator. Thereafter, 50 *μ*l of MTT solution was added to each well, and the plate was incubated in a humidified incubator in 5% CO_2_ for an additional 3 h at 37°C. After incubation, the medium was removed and dimethyl sulfoxide (DMSO) (100 *μ*l per well) was added into each well, and the plate was again incubated at 37°C and 5% CO_2_ on a gentle shaker for 10 min. The plates were gently swirled for 10 min at room temperature to dissolve the precipitate, and the optical density was measured using a microplate reader (Thermo Scientific, USA) at 550 nm. The negative control wells contained only untreated cells and all experiments were performed in triplicate.

The inhibition percentages were determined using the formula

(2) $$\begin{array}{c} \text{viability}\left(\%\right)=\frac{\text{average absorbance of test }\left(\text{treated cells}\right)}{\text{average absorbance of control }\left(\text{untreated cells}\right)}\;\times 100\%. \end{array}$$

The percentage inhibition values were plotted versus extract concentrations to yield sigmoidal dose-response curves from which the 50% cytotoxic concentrations (IC_50_) values were determined with GraphPad Prism version 8.0.0 for Windows (GraphPad Software, San Diego, California, USA, http://www.graphpad.com).

### 2.7. Cell Viability Assay of Fraction N97 against Normal HFF Cells

The cytotoxicity effect of N97 on mammalian cell HFF (human foreskin fibroblasts) cells was performed according to a standard protocol [21]. In brief, the HFF cells were cultured in a complete medium containing 13.5 g/l DMEM, 10% (v/v) foetal bovine serum, 0.2% (w/v) sodium bicarbonate (Sigma), and 50 *μ*g/ml gentamicin. The cells (5 × 10^3^ cells/100 *μ*l/well) in complete medium were seeded into 96-well flat-bottom tissue culture plates. After 24 h incubation, 80 *μ*l of various concentrations of extract solution were added to each well and the plate was incubated for 48 h in a humidified atmosphere at 37°C and 5% CO_2_. DMSO (0.4% v/v) was tested as the positive control. Following incubation, 20 *μ*l of a novel tetrazolium compound (3-(4,5-dimethylthiazol-2-yl)-5-(3-carboxymethoxyphenyl)-2-(4-sulfophenyl)-2H-tetrazolium, inner salt; MTS) and an electron coupling reagent (phenazine methosulfate; PMS) (Promega CellTiter 96® AQ_ueous_ Nonradioactive Cell Proliferation Assay) was added to each well, gently mixed, and incubated for another 1.5 h at 37°C. Thereafter, the supernatant was carefully removed and 100 *µ*l DMSO (quench agent) was added to the cell pellets to dissolve the formazan crystals produced. The formazan solution was measured by recording the optical density (OD) in each well using a microtiter plate reader (Biotek EL800, USA) at 490 nm.

Mean ODs were used to calculate the percent growth inhibition of HFF cells by extracts using the following formula:

(3) $$\begin{array}{c} \text{growth inhibition}\left(\%\right)=\frac{\text{OD}c-\text{OD}t}{\text{OD}c}\times 100\%, \end{array}$$

where OD = optical density; *c* = control (cells only); and *t* = test (cells + extract).

The 50% inhibitory concentration (IC_50_) and the 50% cytotoxic concentration (CC_50_) values were determined as described above.

### 2.8. Selectivity Index (SI)

The selective toxicity of the N97 fungi extract towards the HeLa cell line, relative to the HFF noncancerous cell line, was expressed as the selectivity index (SI) [22]:

(4) $$\begin{array}{c} \text{selectivity index}\left(\text{SI}\right)=\frac{{\text{CC}}_{50}\text{ in noncancer cell line}\left(\text{HFF cells}\right)}{{\text{IC}}_{50}\text{ in cancer cell line}\left(\text{HeLa cells}\right)}\times 100\%. \end{array}$$

### 2.9. Annexin V/PI Mechanism Action Studies of Plant Extracts for Apoptosis or Necrosis Induction

The promising N97 fungal extract was selected for apoptosis determination using HeLa cells. Labelling of early apoptotic and dead cells of N97 extract was performed according to the manufacturer's instructions from the Alexa Fluor 488 Annexin V/Dead Cell Apoptosis Kit (Thermo Fisher Scientific Inc., Germany) and samples were analysed using flow cytometry. In brief, the HeLa cells were cultured to a confluence of 80–90% in Iscove's Modified Dulbecco's Medium (IMDM) supplemented with 10% HIFBS. The cells were trypsinized and suspensions of 1 × 10^6^ cells/ml were plated into a six-well microplate and incubated in a humidified atmosphere at 37°C and 5% CO_2_. Following incubation, the cells were treated with different concentrations of the extract, and the plate was incubated at 37°C and 5% CO_2_ for another 48 h. After the incubation time, the changes in treated cells were observed microscopically and compared to the untreated control sample. After the required observations were recorded, the cells were treated with Trypsin-EDTA (300 *µ*l/well) and harvested by centrifugation at 3000 rpm for 10 min. The cells were washed using ice-cold 1X Annexin V binding buffer and kept in the dark for 30 min. Annexin V dye (3.5 *µ*l) was aliquoted into each well, except the unstained well with no dye, and incubated in a darkroom for 30 min at 4°C. Thereafter, 5 *µ*l of propidium iodide (PI) was added to each well and incubated for an additional 30 min. Annexin binding buffer was adjusted in each well to 100 *µ*g/ml and then transferred to a FACS tube to measure fluorescence via flow cytometry. The percentage of apoptotic and necrotic cells were determined using GraphPad Prism version 8.0.0 for Windows, GraphPad Software, San Diego, California, USA, http://www.graphpad.com.

### 2.10. Statistics Analysis

Data collected from at least three independent experiments were analysed using One-Way ANOVA using Graph Pad Prism. Data are expressed as mean ± SD of experiments performed in triplicate. Error bars represent the SD and ^*∗*^*p* < 0.05, ^*∗∗*^*p* < 0.001, ^*∗∗∗*^*p* < 0.0001, significant difference compared to untreated sample.

## 3. Results

### 3.1. Isolation and Fermentation of Endophytes

**Figure 1:**
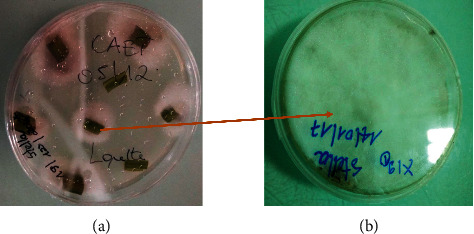
Culture of leave fragments of *T. catappa* on potato dextrose agar (PDA) plates showing emerging mycelia of endophytic fungi (a) after 6 days of incubation and the resulting pure culture of that fungus obtained after subculture on a new PDA plate (b). The culture of leaves from *T. catappa* is an example to illustrate the isolation of endophytes from various parts of the plant. Similar cultures were done for the other parts of *T. catappa* to isolate the different endophytes extracts reported in Table 1.

Highly sterile conditions were maintained for the isolation of endophytes (Figure 1). The conditions used to minimize contamination during isolation were as follows:

- Fresh parts from healthy plants were carefully sampled for isolation work. Short-term preservation and postharvest measures were carried out to avoid contamination.
- The plates were examined daily to ensure that the observed growth sprung from the inner part of the samples. In cases of surface growth, plates containing the sample were discarded.
- The imprint plates were examined for sterility and the whole batch was discarded if any growth was observed on the imprint plate.
- Each 500 ml flask was first autoclaved to avoid any contamination before the initiation of endophytic fungal cultures.

**Figure 2:**
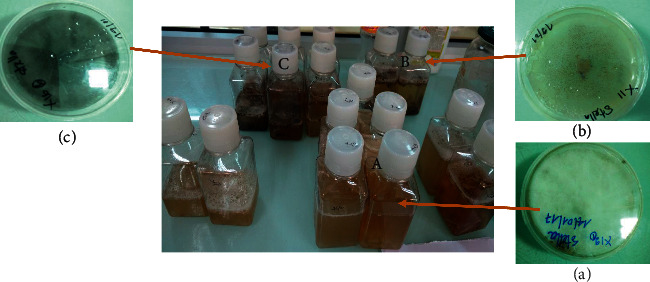
Fermentation procedure of the isolated strains in PDA broth at 25°C for 14 days. (A) Endophyte from stem bark of *T. catappa* (B) Endophyte from leaves of *C. odorata*, (C) Endophyte from leaves of *T. mantaly*.

Following the isolation of endophytic fungal sprouts from 10-day-old cultures in PDA agar Petri dishes, fermentations of endophytic fungal cultures were initiated by transferring five 6 mm agar plugs of each fungus into sterile 500 ml of PDA broth medium and incubating the suspensions 25 ± 2°C under static conditions for 14 days (Figure 2). A flask of rice medium without any inoculum served as a control or some of them were cultured in PDA broth.

### 3.2. Extraction of Metabolites from Endophytic Fungi

The results of the extraction of the metabolites from the endophytic fungi are reported in Table 1 and are similar to that obtained elsewhere [23].

### 3.3. Cytotoxicity Profile of Endophytic Extracts against HeLa Cells

The ability of endophytic fungal extracts to inhibit HeLa cell growth was determined using the MTT assay. Most of the extracts decreased HeLa cell viability in a concentration-dependent manner, with the exception of N8; anticancer activity could not be determined because the concentration was too high (Supplementary materials, Figures S1–S6). However, all the other extracts exhibited negligible anticancer activity. The percentage inhibition ranged between 33.35 and 149.2 *µ*g/ml. IC_50_ values of 33.55 *µ*g/ml, 136.8 *µ*g/ml, 149.2 *µ*g/ml, and 175.8 *µ*g/ml were obtained for N97, N223, N169, and N2 extracts, respectively. N97 was the most active extract with an IC_50_ of 33.35 *µ*g/ml (Table 2). The N7 extract activity could not be determined due to low yield. The disparities in the activity may be due to different locations and amounts of bioactive compounds isolated from each endophytic fungus. A previous report indicated that secondary plant metabolites could vary according to location and storage organs [24].

### 3.4. Cytotoxicity Effect of N97 against the Normal HFF Cells

The (3-(4,5-dimethylthiazol-2-yl)-5-(3-carboxymethoxyphenyl)-2-(4-sulfophenyl)-2H-tetrazolium, inner salt; MTS) and an electron coupling reagent (phenazine methosulfate; PMS) (Promega CellTiter 96® AQ_ueous_ Nonradioactive Cell Proliferation Assay) was used to check the safety of the most active endophyte extract, N97, against HFF cells. This extract exhibited a cytotoxic concentration (CC_50_) of 268.4 *µ*g/ml against normal human foreskin fibroblast (HFF) cells, with an IC_50_ of 33.35 *µ*g/ml against the HeLa cervical cancer cell line and the corresponding selectivity index (SI) was determined to be 8.05 (Table 3). The SI of 8.05 obtained with N97 in HeLa cells implies that the extract can be considered less toxic to normal HFF cells and the extract will preferentially destroy the HeLa cancer cells and to a lesser degree the HFF cells, if both cell lines were to be mixed with the same extract.

### 3.5. Morphological Observation after 48 h of Incubation

**Figure 3:**
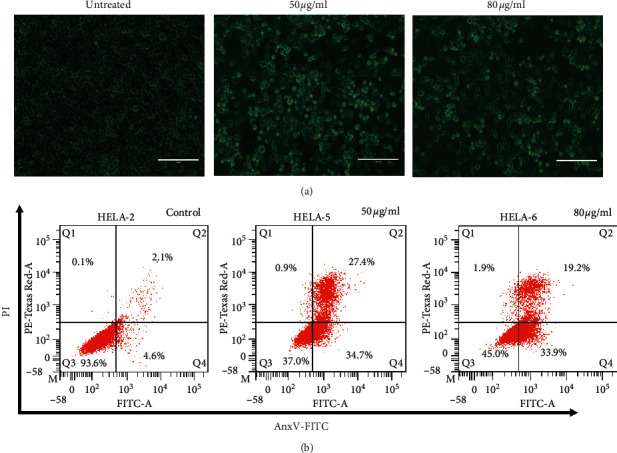
Representative photomicrographs of the cellular morphological signatures after treatments with N97. Apoptosis was detected by FCM Annexin V/PI staining (a). Representative Annexin V FITC-A vs propidium iodide-A contour plots from three concentrations of N97. HeLa cells were treated with the N97 extract for 48 h (b).

Microscopic observation showed a higher reduction in cell density, as well as signs of cellular shrinkage and aggregation in the N97-treated cultures compared to the untreated controls (Figure 3(a)). However, N97-treated cells had reduced cell density compared to the untreated controls. The N97-treated cells thus exhibited features associated with cells undergoing apoptosis. The N97 extract induced greater than 50% inhibition mediated through apoptosis of HeLa cells (Figures 3(a) and 3(b)). Moreover, a higher reduction in the percentage of viable cells was observed in the HFF control cells (93.6 ± 0.3), but the percentages of viable HeLa cells were 37 ± 0.05 and 45 ± 0.1, respectively, for the 50 *µ*g/ml and 80 *µ*g/ml N97 extract treatments. Also, the percentages of cell populations were increased in late apoptosis (Annexin+/PI−), with the percentage of 27.4 ± 0.3 and 19.2 ± 0.01 obtained, respectively, for 50 *µ*g/ml and 80 *µ*g/ml of N97 extract, and which is 2.1 ± 0.1 obtained for the control in late apoptosis (Annexin V+/PI+). Additionally, the percentage of 34.7 ± 0.1, 33.9 ± 0.4 were, respectively, obtained following exposure to 50 *µ*g/ml and 80 *µ*g/ml of the N97 extract compared to the control which stood at 4.6 ± 0.2, in early apoptosis (Annexin V+/PI−) (Figure 3(b); Supplementary materials, Table S1). The Annexin V/PI results confirmed that the N97 extract induces the inhibition of HeLa cells through apoptosis. The data indicate that with increasing N97 extract concentrations, there is a concomitant decrease in the number of viable cells. Therefore, the percentage of apoptotic cells after treatment with 50 *µ*g/ml and 80 *µ*g/ml of the extract was drastically increased (*p* < 0.0001).

### 3.6. Mechanisms of Action of N97 Extract via Annexin V/PI

**Figure 4:**
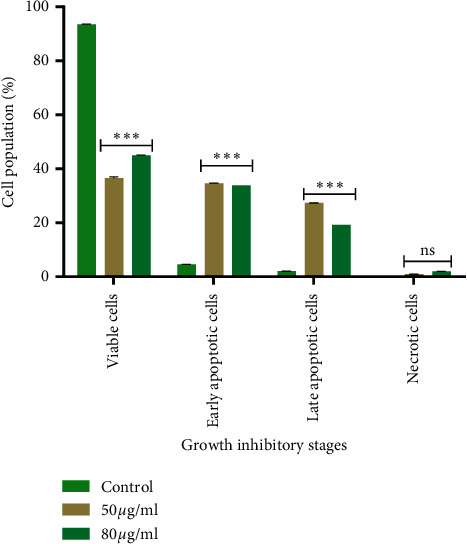
Apoptotic effect of N97 against HeLa cells. The proportions of Annexin V+/PI− and Annexin V+/PI + cells indicating the percentage of viable cells, early and late stages of apoptosis, and necrosis. A two-tailed, unpaired *t*-test was used to analyse significance. ^*∗*^*p* < 0.05, ^*∗∗*^*p* < 0.001, ^*∗∗∗*^*p* < 0.0001, significant difference compared to the untreated sample.

Apoptosis is a highly organized cell death process characterized by the loss of plasma membrane asymmetry, condensation of nuclear chromatin, nuclear disintegrations, and DNA cleavage by enzymes [25]. Agents that suppress the proliferation of cancer cells by inducing apoptosis may represent a useful mechanistic approach to both cancer chemotherapy and preventing unfavourable side effects and drug resistance [26]. In this study, the most specific active endophytic fungal extract, N97, was evaluated for its potential to induce apoptosis in HeLa cells using the Annexin/PI Apoptosis Detection kit and the results are summarized in the supplementary material (Table S1). FACScan analysis of the untreated cells revealed that the cells were negative for Annexin V-FITC and PI (Figure 3(b); Supplementary materials, Table S1), indicating that they were viable and not undergoing apoptosis. Cells that have entered the necrotic phase are positive for PI only, thus the HeLa cells treated with the N97 endophyte extract for 24 h revealed some morphological features, and the percentage of apoptosis cells varied in a concentration-dependent manner as illustrated in Figures 3 and 4. A remarkable observation was that endophyte extract N97 at 50 *µ*g/ml resulted in the lowest number of viable cells (37.0%) and was able to induce 34.7% and 27.4% early and late apoptotic cell death, respectively. Moreover, endophyte 80 *µ*g/ml of extract N97 induced 33.9% and 19.2% early and late apoptotic cell death, respectively, in human cervical HeLa cancer cells (Figure 4). The results strongly support the ability of the endophyte N97 extract to trigger HeLa cell death via apoptosis.

## 4. Discussion

Currently, finding new, powerful, effective, and affordable anticancer drugs is a challenge. Here, we report the anticancer activity and apoptosis-inducing effects of endophytic extracts from the medicinal plant *Terminalia catappa* against HeLa cells. In addition, the cytotoxic effects of endophytic extract N97 were also evaluated in nontumour HFF cells to determine the possible differences in responses to extracts between cancer and normal cells. For the antiproliferative test, we used the MTT reagent and within 48 h of exposure, the extracts at a concentration of 100 *μ*g/ml had demonstrated potency to reduce cell viability by more than 90% in a dose-dependent manner. Of all the fungal extracts tested, the N97 extract from *T. catappa* exhibited the most promising results with an IC_50_ value of 33.35 *µ*g/ml against HeLa cells. Accordingly, the safety of extract N97 was checked on normal HFF cells. A CC_50_ of 268.4 *µ*g/ml and a selectivity index (SI) of 8.01 were obtained in HFF cells. It has been proven that extracts from endophytic fungi contain compounds with anticancer proprieties [27].

Thus, the observed cytotoxicity reported in this study can be attributed to the compounds present in the fungal extracts. Indeed, many researchers have confirmed that the extracts from endophytes are excellent producers of strong cytotoxic metabolites [28]. According to the United States National Cancer Institute Plant Screening Program, a crude extract is generally considered to have *in vitro* cytotoxic activity if the IC_50_ values is <30–40 *μ*g/ml [29]. The N97 extract can, therefore, be regarded as a mixture with cytotoxic potential. Our study demonstrated that the noncancerous cell HFF line was more resistant to the effects of the N97 extract, but toxic to the cervical HeLa cancerous cells. This selective toxicity towards cancer cells is a characteristic that is highly sought after in the development of new anticancer treatments because much of the adverse effects observed with anticancer drugs result from toxicity to normal cells [30]. The higher SI obtained show that the fungal extract is more toxic to HeLa cells than to normal HFF cells and also highlights the relative safety of N97. Our results confirm that the endophytic fungi residing in the stem bark of *Terminalia catappa* can inhibit the cell viability and growth of HeLa cell lines.

Furthermore, the results also indicated that the N97 extract does not specifically affect the proliferation of normal cells. To check how N97 induces HeLa cell death, the apoptosis induction assay was performed using the Annexin V/PI. Our microscopic observations illustrated the characteristic morphological features of cells undergoing apoptosis (higher reduction of the number of cells, shrinkage, and aggregation of cells accompanied by DNA fragmentation). Chemotherapeutic drugs kill tumour cells by activating a cascade of events resulting in apoptosis [31]. Our study agrees with this line of thought as we provide evidence that the N97 extract did not induce necrosis in HeLa cells because, the necrotic cells appeared in a very small percentage of the cell population analysed. However, the N97 extracts were able to induce more than 50% inhibition of cell growth, mediated through apoptosis of HeLa cells, with the classic features of apoptosis, including initiator and/or effector caspase activation and internucleosomal DNA fragmentation. Moreover, the increase of the apoptotic population could be p53 protein-dependent and lead to the activation of caspases, especially effector caspase-3.

## 5. Conclusion

In this study, the N97 endophytic fungal extract exhibited a significant antiproliferative effect on HeLa cells. The growth inhibitory potential of the N97 extract on HeLa cells was confirmed to be mediated via apoptosis. Therefore, further research to isolate and identify the bioactive compounds responsible for the specified biological activities and their possible mechanisms of action is currently being pursued by our research group. To the best of our knowledge, the demonstrated anticancer activity of extract N97 in this study is reported here for the first time.

## Figures and Tables

**Table 1:** The different metabolites extracted from the endophytic fungi and their corresponding plant material.

Codes	Organs	Endophytes	Microorganisms
N97	Stem bark	Endophytic extracts from stem bark of *Terminalia catappa*	*Trichoderma sp*
N7	Flowers	Endophytic extracts from flowers of *Cananga odorata*	*Phoma sp*
N169	Twigs	Endophytic extracts from twigs of *Terminalia mantaly*	*Phomopsis phyllanticola*
N2	Stems	Endophytic extracts from stem of *Cananga odorata*	*Fusarium oxysporum*
N8	Leaves	Endophytic extracts from leaves of *Cananga odorata*	*Collectotrichum sp*
N233	Leaves	Endophytic extracts from leaves of *Terminalia mantaly*	*Cryptococcus flavescens*

N97: endophytic extracts from stem bark of *Terminalia catappa*; N169: endophytic extracts from twigs of *Terminalia mantaly*; N2: endophytic extracts from stem of *Cananga odorata*; N8: endophytic extracts from leaves of *Cananga odorata*; N233: endophytic extracts from leaves of *Terminalia mantaly*.

**Table 2:** Anticancer screening of endophytic fungi extracts on uterine cervical cancer cells (HeLa). The anticancer activity was determined via MTT assay after exposure to metabolite extracts for 48 h.

Code	HeLa cells
	IC_50_ (*µ*g/ml)
N8	NC
N97	33.35
N223	136.80
N169	149.20
N2	175.80
N7	NC

The IC_50_ values were determined using GraphPad Prism. ^*∗*^*p* < 0.05, ^*∗∗*^*p* < 0.001, ^*∗∗∗*^*p* < 0.0001 significantly different from control. Samples were compared using One-Way ANOVA and Tukey comparison of all pairs of columns. IC_50_: extract concentration required to reduce HeLa cancer cell line by 50%. N97: endophytic extracts from stem bark of *Terminalia catappa*; N169: endophytic extracts from twigs of *Terminalia mantaly*; N2: endophytic extracts from stem of *Cananga odorata*; N8: endophytic extracts from leaves of *Cananga odorata*; N233: endophytic extracts from leaves of *Terminalia mantaly*. NC: none converged.

**Table 3:** Cytotoxicity effect of N97 against HFF cells.

Code	Noncancerous fibroblast HFF cells	
	CC_50_ (*µ*g/ml)	SI
N97	268.4	8.05

CC_50_: extract concentration required to reduce HFF cell viability by 50%; SI (selectivity index) = CC_50_/IC_50_. CC_50_ estimates were determined using GraphPad Prism. ^*∗*^*p* < 0.05, ^*∗∗*^*p* < 0.001, ^*∗∗∗*^*p* < 0.0001 significantly different compared to control. Samples were compared using One-Way ANOVA and Tukey comparisons of all pairs of columns. N97: endophytic extracts from stem bark of *Terminalia catappa*.

## Data Availability

The data used to support the findings of the present study are available from the corresponding author upon request.

## Supplementary Materials

Supplementary Materials Figure S1: Effects of increasing concentrations of the N8 endophytic extract on the viability of HeLa cells. Cell viability after 24 h exposure to the extract was analysed using the MTT assay. Results represent the average of two independent experiments performed in triplicate. A two-tailed, unpaired *t*-test was used to analyse significance. ^*∗*^*p* < 0.05, ^*∗∗*^*p* < 0.001, ^*∗∗∗*^*p* < 0.0001, significant difference compared to untreated sample. Figure S2: Effects of increasing concentrations of the N97 endophytic extract on the viability of HeLa cells. Cell viability after 24 h exposure to the extract was analysed by the MTT assay. Results represent the average of two independent experiments performed in triplicate. A two-tailed, unpaired *t*-test was used to analyse significance. ^*∗*^*p* < 0.05, ^*∗∗*^*p* < 0.001, ^*∗∗∗*^*p* < 0.0001, significant difference compared to untreated sample. Figure S3: Effects of increasing concentrations of the N223 endophytic extract on the viability of HeLa cells. Cell viability after 24 h exposure to the extract was analysed using the MTT assay. Results represent the average of two independent experiments performed in triplicate. A two-tailed, unpaired *t*-test was used to analyse significance. ^*∗*^*p* < 0.05, ^*∗∗*^*p* < 0.001, ^*∗∗∗*^*p* < 0.0001, significant difference compared to untreated sample. Figure S4: Effects of increasing concentrations of N169 endophytic extract on the viability of HeLa cells. Cell viability after 24 h exposure to the extract was analysed using the MTT assay. Results represent the average of two independent experiments performed in triplicate. A two-tailed, unpaired *t*-test was used to analyse significance. ^*∗*^*p* < 0.05, ^*∗∗*^*p* < 0.001, ^*∗∗∗*^*p* < 0.0001, significant difference compared to untreated sample. Figure S5: Effects of increasing concentrations of N2 endophytic extract on the viability of HeLa cells. Cell viability after 24 h exposure to the extract was analysed using the MTT assay. Results represent the average of two independent experiments performed in triplicate. A two-tailed, unpaired *t*-test was used to analyse significance. ^*∗*^*p* < 0.05, ^*∗∗*^*p* < 0.001, ^*∗∗∗*^*p* < 0.0001, significant difference compared to untreated sample. Figure S6: effects of increasing concentrations of N7 endophytic extract on the viability of HeLa cells. Cell viability after 24 h exposure to the extract was analysed using the MTT assay. Results represent the average of two independent experiments performed in triplicate. A two-tailed, unpaired *t*-test was used to analyse significance. ^*∗*^*p* < 0.05, ^*∗∗*^*p* < 0.001, ^*∗∗∗*^*p* < 0.0001, significant difference compared to untreated sample. Table S1: Annexin V/PI results of N-97 with HeLa cells. (Annexin V−/PI+, left upper quadrant): necrotic cells; (Annexin V+/PI−, right lower quadrant): early apoptotic cells; (Annexin V+/PI+, right upper quadrant): late apoptotic cells; (Annexin V−/PI−, left lower quadrant): viable cells.
