# Supplementary material for: Cytotoxicity Potential of Endophytic Fungi Extracts from Terminalia catappa against Human Cervical Cancer Cells
Source: J Toxicol. 2020 Sep 22;2020:8871152. doi: 10.1155/2020/8871152 (PMC7528136; doi:10.1155/2020/8871152)
Supplement: Supplementary Materials — Figure S1: Effects of increasing concentrations of the N8 endophytic extract on the viability of HeLa cells. Cell viability after 24 h exposure to the extract was analysed using the MTT assay. Results represent the average of two independent experiments performed in triplicate. A two-tailed, unpaired t-test was used to analyse significance. ∗p < 0.05, ∗∗p < 0.001, ∗∗∗p < 0.0001, significant difference compared to untreated sample. Figure S2: Effects of increasing concentrations of the N97 endophytic extract on the viability of HeLa cells. Cell viability after 24 h exposure to the extract was analysed by the MTT assay. Results represent the average of two independent experiments performed in triplicate. A two-tailed, unpaired t-test was used to analyse significance. ∗p < 0.05, ∗∗p < 0.001, ∗∗∗p < 0.0001, significant difference compared to untreated sample. Figure S3: Effects of increasing concentrations of the N223 endophytic extract on the viability of HeLa cells. Cell viability after 24 h exposure to the extract was analysed using the MTT assay. Results represent the average of two independent experiments performed in triplicate. A two-tailed, unpaired t-test was used to analyse significance. ∗p < 0.05, ∗∗p < 0.001, ∗∗∗p < 0.0001, significant difference compared to untreated sample. Figure S4: Effects of increasing concentrations of N169 endophytic extract on the viability of HeLa cells. Cell viability after 24 h exposure to the extract was analysed using the MTT assay. Results represent the average of two independent experiments performed in triplicate. A two-tailed, unpaired t-test was used to analyse significance. ∗p < 0.05, ∗∗p < 0.001, ∗∗∗p < 0.0001, significant difference compared to untreated sample. Figure S5: Effects of increasing concentrations of N2 endophytic extract on the viability of HeLa cells. Cell viability after 24 h exposure to the extract was analysed using the MTT assay. Results represent the average of two independent experiments performed in [file 8871152.f1.docx]

**Supplementary Material Data**

**Figure S1: Effects of increasing concentrations of the N8 endophytic extract on the viability of HeLa cells**. Cell viability after 24 h exposure to the extract was analysed using the MTT assay. Results represent the average of two independent experiments performed in triplicate. A two-tailed, unpaired t-test was used to analyse significance. *p<0.05, ** p<0.001, ***p<0.0001, significant difference compared to untreated sample.

**Figure S2: Effects of increasing concentrations of N97 endophytic extract on the viability of HeLa cells**. Cell viability after 24 h exposure to the extract was analysed using the MTT assay. Results represent the average of two independent experiments performed in triplicate. A two-tailed, unpaired t-test was used to analyse significance. *p<0.05, ** p<0.001, ***p<0.0001, significant difference compared to untreated sample.

**Figure S3: Effects of increasing concentrations of the N223 endophytic extract on the viability of HeLa cells**. Cell viability after 24 h exposure to the extract was analysed using the MTT assay. Results represent the average of two independent experiments performed in triplicate. A two-tailed, unpaired t-test was used to analyse significance. *p<0.05, ** p<0.001, ***p<0.0001, significant difference compared to untreated sample.

**Figure S4: Effects of increasing concentrations of N169 endophytic extract on the viability of HeLa cells**. Cell viability after 24 h exposure to the extract was analysed using the MTT assay. Results represent the average of two independent experiments performed in triplicate. A two-tailed, unpaired t-test was used to analyse significance. *p<0.05, ** p<0.001, ***p<0.0001, significant difference compared to untreated sample.

**Figure S5: Effects of increasing concentrations of N2 endophytic extract on the viability of HeLa cells**. Cell viability after 24 h exposure to the extract was analysed using the MTT assay. Results represent the average of two independent experiments performed in triplicate. A two-tailed, unpaired t-test was used to analyse significance. *p<0.05, ** p<0.001, ***p<0.0001, significant difference compared to untreated sample.

**Figure S6: Effects of increasing concentrations of N7 endophytic extract on the viability of HeLa cells**. Cell viability after 24 h exposure to the extract was analysed using the MTT assay. Results represent the average of two independent experiments performed in triplicate. A two-tailed, unpaired t-test was used to analyse significance. *p<0.05, ** p<0.001, ***p<0.0001, significant difference compared to untreated sample.

**Table S1: Annexin V/PI results of N-97 with HeLa cells**.

| **Different stages** | **Unstained** | **Control** | **Dose (****µg/ml)** | |
| --- | --- | --- | --- | --- |
|  |  |  | **50** | **80** |
| LL or Annexin V-/PI- | 98.4 ± 0.5^c^ | 93.6 ± 0.3^c^ | 37 ± 0.05^a^ | 45 ± 0.1^b^ |
| LR or Annexin V+/PI- | 0.1 ± 0.2^a^ | 4.6 ± 0.2^b^ | 34.7 ± 0.1^c^ | 33.9 ± 0.4^c^ |
| UL or Annexin V-/PI+ | 1.3 ± 0.5^b^ | 0.1 ± 0.02^a^ | 0.9 ± 0.1^a^ | 1.9 ± 0.5^b^ |
| UR or Annexin V+/PI+ | 0.3 ± 0.4^a^ | 2.1 ± 0.1^b^ | 27.4 ± 0.3^d^ | 19.2 ± 0.01^c^ |
| **Legend**: (Annexin V-/PI+, left upper quadrant): necrotic cells; (Annexin V+/PI-, right lower quadrant): early apoptotic cells; (Annexin V+/PI+, right upper quadrant): late apoptotic cells; (Annexin V-/PI-, left lower quadrant): viable cells. | | | | |
